# Seroepidemiology of *Coxiella burnetii* in Domestic and Wild Ruminant Species in Southern Spain

**DOI:** 10.3390/ani14213072

**Published:** 2024-10-24

**Authors:** Débora Jiménez-Martín, Javier Caballero-Gómez, David Cano-Terriza, Saúl Jiménez-Ruiz, Jorge Paniagua, Paloma Prieto-Yerro, Sabrina Castro-Scholten, Ignacio García-Bocanegra

**Affiliations:** 1 Grupo de Investigación en Sanidad Animal y Zoonosis (GISAZ), Departamento de Sanidad Animal, UIC Zoonosis y Enfermedades Emergentes ENZOEM, Universidad de Córdoba, 14071 Córdoba, Spain; debora.djm@gmail.com (D.J.-M.); javiercaballero15@gmail.com (J.C.-G.); saul.jimenez.ruiz@gmail.com (S.J.-R.); jorgepaniaguarisueno@gmail.com (J.P.); sabrina1996cs@gmail.com (S.C.-S.); nacho.garcia@uco.es (I.G.-B.); 2 Grupo de Virología Clínica y Zoonosis, Unidad de Enfermedades Infecciosas, Instituto Maimónides de Investigación Biomédica de Córdoba (IMIBIC), Hospital Universitario Reina Sofía, Universidad de Córdoba, 14014 Córdoba, Spain; 3 CIBERINFEC, ISCIII-CIBER de Enfermedades Infecciosas, Instituto de Salud Carlos III, 28029 Madrid, Spain; 4 Parque Natural Sierras de Cazorla, Segura y Las Villas, Junta de Andalucía, 23470 Cazorla, Spain; palomam.prieto@juntadeandalucia.es

**Keywords:** goat, Q fever, sheep, wild ruminants, zoonoses

## Abstract

The European Food Safety Authority has recently listed Q fever as a priority for setting up a coordinated surveillance system. Although Spain is the country with the highest human incidence of the disease in the European Union, updated data on *Coxiella burnetii* in ruminants are still limited. A total of 780 serum samples from small ruminants and 605 sera from wild ruminants were collected in the Mediterranean ecosystems of southern Spain during the period 2015–2023. Anti-*C. burnetii* antibodies were detected using a commercial indirect ELISA. The overall individual seroprevalence in the small ruminants was 49.1% (383/780; 95% CI: 45.6–52.6). Antibodies against *C. burnetii* were identified in 40.0% (156/390) of sheep and in 58.2% (227/390) of goats. At least one seropositive animal was observed in all sheep (100%) and in 92.3% of goat flocks. The species (goat) and the existence of reproductive disorders in primiparous females were potential risk factors for *C. burnetii* exposure in small ruminant farms. In the wild ruminants, the overall seroprevalence against *C. burnetii* was 1.5% (9/605; 95% CI: 0.8–2.8). Anti-*C. burnetii* antibodies were found in 1.8% (2/110) of mouflon, 1.5% (6/390) of red deer, and 1.0% (1/105) of Iberian ibex. The high exposure of the small ruminants to *C. burnetii*, particularly in goats, detected in the present study is of animal and public health concern. Our results denote that wild ruminants only play a minor role in the epidemiology of this bacterium in southern Spain and suggest an independent epidemiological cycle of *C. burnetii* in domestic and wild ruminant species in the study area.

## Simple Summary

Q fever is a multi-host zoonotic disease of animal and public health concern, with ruminants being its main reservoirs. In the present study, we aimed to determine the seroprevalence and to identify risk factors for the exposure to *Coxiella burnetii* in domestic (390 sheep and 390 goats) and wild ruminants (390 red deer, 110 mouflon, and 105 Iberian ibex) in the Mediterranean ecosystems of southern Spain. The overall individual seroprevalence in the small ruminants was 49.1% (383/780). At least one seropositive animal was observed in all sheep (100%) and in 92.3% of goat flocks. The species (goat) and the existence of reproductive disorders in primiparous females were potential risk factors for *C. burnetii* exposure in small ruminant farms. In wild ruminants, the overall seroprevalence against the pathogen was 1.5% (9/605). The high exposure of the small ruminants to *C. burnetii*, particularly in goats, detected in the present study is of animal and public health concern. Our results denote that wild ruminants only play a minor role in the epidemiology of this bacterium in southern Spain and suggest an independent epidemiological cycle of *C. burnetii* in domestic and wild ruminant species in the study area.

## 1. Introduction

Q fever is an important globally distributed zoonosis with a major impact on public and animal health [1,2]. The disease is caused by the small intracellular bacterium *Coxiella burnetii,* which is listed in the category B as a bioterrorism agent due to its significant stability, resistance, aerosolization capability, and high virulence [3,4,5]. In recent years, more than 700 human cases of Q fever have been recorded annually in Europe [6], with Spain reporting the highest number of them since 2017 [6,7]. The clinical spectrum of this disease in humans encompasses flu-like illness, high fever, myalgia, headache, muscle pain, and potentially fatal endocarditis, among other symptoms [8]. Humans mainly acquire *C. burnetii* infections zoonotically by inhaling contaminated dust with birth materials, urine, or feces from infected animals [6]. Furthermore, infections from contaminated food or unpasteurized milk, as well as tick-borne transmissions, have been reported or suggested [9,10,11]. Given this scenario, the European Food Safety Authority (EFSA) has recently listed this disease as a priority for implementing a coordinated One Health-based surveillance system [12].

Domestic ruminants are the main natural reservoirs of *C. burnetii*, with sheep and goats being the major hosts [13,14]. Accordingly, the vast majority of human Q fever outbreaks are epidemiologically linked to small ruminants, underscoring their critical role in the zoonotic transmission of *C. burnetii* [13,15]. In addition, this disease exerts a significant economic burden in small ruminant production systems, since it is associated with production losses and the costs of implementing vaccination and control programs [16,17]. Currently, only one inactivated vaccine is approved for use in small ruminants [18]. When it is combined with management and biosecurity practices, farms can significantly reduce *C. burnetii* infections [19], which usually cause reproductive disorders such as late abortions, metritis, stillbirths, weak offspring, or infertility [1,13,20,21]. Besides livestock, this multi-host bacterium has also been reported in different wild ruminant species, suggesting their potential involvement in the sylvatic cycle of *C. burnetii* [22,23]. Thus, the circulation of *C. burnetii* in wild ruminants could imply a risk of infection to other sympatric species, including humans [22,24,25,26].

The high incidence of Q fever in humans across Europe reflects the circulation of the bacterium in animal reservoirs [14,22] and highlights the need to monitor the bacterium in ruminants to mitigate the zoonotic threat. However, given that epidemiological contexts are different [e.g., seroprevalences vary widely in goat and sheep in Europe from less than 0.5% to more than 75%] [10], the associated public health risk varies accordingly.

Spain accounts for the largest census of small domestic ruminants in the European Union (EU) [27,28] and the densities of wild ruminant populations have considerably increased in this country throughout the last few decades [29,30]. Of note, Andalusia (southern Spain), a region that covers an area of 87,591 km^2^, recorded the highest number of Q fever human cases reported in recent years in mainland Spain [31] and hosts one of the largest populations of small and wild ruminants in this country [30,32]. Although the circulation of *C. burnetii* has already been reported in small domestic and wild ruminants in certain regions of Spain [33,34,35,36,37,38,39,40] to date, the information about the exposure of *C. burnetii* in these species is still very scarce and only focused on geographically limited areas. Therefore, our objectives were to evaluate the seroprevalence and risk factors associated with *C. burnetii* exposure in sheep, goats, and wild ruminant species [red deer (*Cervus elaphus*), mouflon (*Ovis aries musimon*), and Iberian ibex (*Capra pyrenaica*)] in southern Spain.

## 2. Materials and Methods

### 2.1. Study Design

A cross-sectional study was carried out on goat and sheep farms in Andalusia (36° N–38°60′ N, 1°75′ W–7°25′ W) to estimate the seroprevalence of *C. burnetii*. The study area is characterized by the Dehesa agroforestry system, interspersed with Mediterranean forests. These areas support a mix of land uses, including agriculture, livestock farming, and hunting. The climate is characterized as continental thermo-Mediterranean, featuring hot, dry summers and mild winters.

The number of sheep and goat samples to be analyzed was determined assuming an estimated prevalence of 50% (which ensures the largest sample size in studies with an unknown prevalence), using a 95% confidence interval (95% CI) and an accepted error of 5% [41]. Stratified sampling by province was performed based on the proportion of sheep and goats present in each province. Fifteen animals from each herd, selected by systematic random sampling, were sampled in order to detect exposure to *C. burnetii* with a 95% probability, assuming a minimum expected seroprevalence of 20%. Finally, between 2015 and 2017, blood samples were collected from 390 sheep and 390 goats from 52 farms (26 sheep and 26 goat farms). The blood samples were taken from the jugular vein using sterile tubes without any anticoagulant and kept refrigerated until their reception at the laboratory. Epidemiological data on the sampled animals and farms were compiled using a standardized questionnaire during personal interviews with the farmers (Table S1 and Supplementary Information). None of the farms involved in the study had vaccinated their animals against *C. burnetii*.

Additionally, between the 2015/2016 and 2022/2023 hunting seasons, a total of 605 wild ruminants were sampled in 32 hunting estates from the study area. Blood samples from hunted red deer (*n* = 390), mouflon (*n* = 110), and Iberian ibex (*n* = 105) were obtained. All the wild ruminants were legally harvested by hunters or culled as part of population control programs in game reserves. The blood was obtained by the puncture of the endocranial venous sinuses [42] and collected into sterile tubes without anticoagulant. Whenever possible, data on the sampled wild ruminant populations, including the location, sampling year, sex, and age (yearlings, sub-adults, or adults) [43] were recorded for each animal. All the sera from the blood samples included in the study were collected by centrifuging them at 400× *g* for 10 min and subsequently kept at −20 °C until the laboratory analysis.

### 2.2. Laboratory Analysis

The presence of antibodies against *C. burnetii* was detected using a commercial indirect ELISA (ID Screen Q Fever Indirect Multispecies^®^, IDVet^®^, Grabels, France). ELISA assays were performed at the Animal Health Laboratory of the University of Córdoba (Spain), according to the manufacturer’s instructions. This multi-species ELISA has been widely used in both domestic and wild ruminant species [44,45,46,47]. The sensitivity and specificity of this assay have been shown to be 100% (IDvet, according to the manufacturer’s internal validation report).

### 2.3. Statistical Analysis

The individual seroprevalence of *C. burnetii* was estimated from the ratio of positive animals to the total number of individuals tested, using the two-sided exact binomial test, with a 95% CI [48]. To identify nonlinear relationships and standardize the scales of the explanatory variables, the continuous variables underwent transformations into qualitative variables, categorized into three groups, with the 33rd and 66th percentiles serving as the cut-offs.

Differences between the *C. burnetii* seroprevalence in the domestic and wild ruminants were assessed using Pearson’s chi-square test. The analysis of the risk factors potentially associated with the exposure to *C. burnetii* in the small domestic and wild ruminants were evaluated separately. Initially, Pearson’s chi-square or Fisher’s exact test was employed, as appropriate, to analyze the associations between the serological results (dependent variable) and the explanatory variables. The variables with a *p*-value < 0.05 in the bivariate analysis were selected for subsequent analyses. Collinearity between pairs of variables was determined by Cramer’s V coefficient. When collinearity was identified (Cramer’s V coefficient ≥ 0.6), the variable with the a priori strongest biological association with *C. burnetii* was retained. Subsequently, the effect of the variables selected in the bivariate analysis was evaluated with a generalized estimating equation (GEE) model. The seropositive animal numbers were assumed to follow a binomial logistic distribution, with flocks as the subject variable. The model was repeated until all the remaining variables were statistically significant (*p*-value < 0.05) and a potential causal effect on the dependent variable existed. The quasi-likelihood information criterion (QIC) was employed to select the most accurate model. SPSS 25.0 software was used to carry out the statistical analyses.

## 3. Results

**Figure 1:**
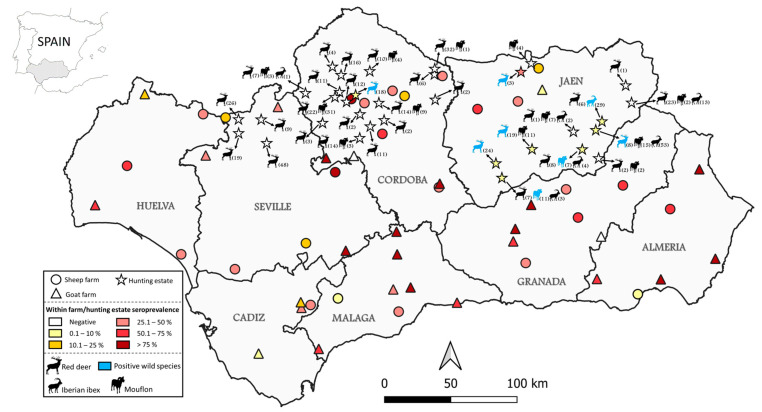
Distribution and seroprevalence of analyzed sheep (circles) and goat (triangles) farms, as well as sampled hunting estates (stars). Color gradients indicate within-farm/hunting estate seroprevalence. At the hunting estate level, seropositive wild ruminants are represented in blue, and the number of sampled individuals is indicated in parentheses next to the silhouette of each species.

The overall individual seroprevalence of *C. burnetii* in the small ruminants was 49.1% (383/780; 95% CI: 45.6–52.6). By species, anti-*C. burnetii* antibodies were detected in 40.0% (156/390; 95% CI: 35.3–44.9) of the sheep and 58.2% (227/390; 95% CI: 53.3–63.0) of the goats. At the farm level, 96.2% (50/52; 95% CI: 87.0–98.9%) of the flocks were *C. burnetii*-seropositive. At least one seropositive animal was detected in all the sheep flocks (100%) and in 24 out of the 26 (92.3%) goat flocks (Figure 1). The within-flock seroprevalence ranged from 6.7% to 86.7% (mean 40.0%) in the sheep and from 6.7% to 100% (mean 63.1%) in the goats. Table S1 shows the explanatory variables gathered from the epidemiological questionnaire and results derived from the bivariate analysis. The final GEE model found two potential risk factors for *C. burnetii* seropositivity in small ruminants: species (goat) and the existence of reproductive disorders in primiparous females (Table 1).

Antibodies against *C. burnetii* were detected in nine out of the six hundred and five (1.5%; 95% CI: 0.8–2.8) wild ruminants tested. By species, the prevalence of antibodies was 1.8% (2/110; 95% CI: 0.5–6.4) in the mouflon, 1.5% (6/390; 95% CI: 0.7–3.3) in the red deer, and 1.0% (1/105; 95% CI: 0.2–5.2) in the Iberian ibex (Table S2). No statistically significant differences were observed in the *C. burnetii* seropositivity among the wildlife species tested (*p* = 0.863). However, the overall seroprevalence in the wild ruminants was significantly lower than that observed in the domestic ruminants (*p* < 0.001). Seropositive animals were detected in eight (25.0%) of the thirty-two hunting estates tested (Figure 1). Additionally, the spatial distribution of the *C. burnetii* exposure was not homogeneous. Significantly higher seropositivity was found in the province of Jaen (3.0%) compared to Cordoba (0.4%) and Seville (0%) (*p* < 0.022). The potential risk factors linked with the exposure to *C. burnetii* in these species were not identified in our multivariate statistical analysis.

## 4. Discussion

Given Q fever has recently been identified by the EFSA as a high-priority disease that requires surveillance, monitoring *C. burnetii* in both domestic and wild ruminants is pivotal for improving coordinated control strategies. This will help to limit the risk of transmission not only between ruminants but also to humans and other wild and domestic sympatric species [12].

The findings obtained in the current study support that small ruminant populations from southern Spain are naturally exposed to *C. burnetii*, which can be of public health concern. The seroprevalence values obtained in sheep (40.0%) and goats (58.2%), were higher than those found in most of the studies previously conducted throughout this country, which have ranged between 1.5 and 31.7% and 6.7 and 60.4% for sheep and goats, respectively [10,33,35,36,37,38,39,40]. Notably, this result aligns with the spatial distribution of Q fever cases in humans across mainland Spain, with the southern region exhibiting the highest incidences [31].

*Coxiella burnetii* is mainly shed during the peri-parturient period through birth products [49], but it can also be excreted in the milk, feces, and urine of infected animals over several weeks [14,50,51]. These secretions and excretions contaminate the environment, where the bacterium can remain viable for months or even years [4,52,53]. Although the detection of anti-*C. burnetii* antibodies does not necessarily indicate active infection or bacterial shedding [51], as some animals can remain seropositive for years post-infection or may not seroconvert during active shedding [54], our results suggest that this bacterium could be widely distributed in farm environments. Recent outbreaks of Q fever in humans in northern Spain were linked to farms with the presence of contaminated dust with *C. burnetii* [55]. Additional epidemiological and molecular studies are warranted to evaluate the zoonotic risk of *C. burnetii* transmission in contaminated environments from small ruminant farms in southern Spain.

Regarding the wild ruminants, the seroprevalences detected in the mouflon (1.8%), red deer (1.5%), and Iberian ibex (1.0%) suggest a limited exposure of these species to *C. burnetii* in the Mediterranean ecosystems of southern Spain. Our results match with those found previously in mouflon and red deer populations from other regions of the Iberian Peninsula, with exposure rates ranging from 1.4 to 6.8% and 1.5 to 8.4%, respectively [25,38,39,47]. On the contrary, previous studies conducted on Iberian ibex showed higher prevalences of anti-*C. burnetii* antibodies (12.6–30.0%) [35,38,56] compared to the 1.0% found in the present study. These observed differences support the need to determine the potential role of wild ruminant species in the sylvatic cycle of *C. burnetii* depending on the different epidemiological scenarios present in the Iberian Peninsula.

The circulation of *C. burnetii* in wild ruminants could potentially contribute to environmental contamination with this bacterium [57]. In addition, direct exposure through handling wild ruminants or game carcasses has proven to be a potential route of zoonotic transmission [26,57]. However, our results suggest a low risk of *C. burnetii* transmission from wild ruminants to humans and other sympatric species within the study area.

Significant differences in the *C. burnetii* seroprevalence were observed between the domestic and wild ruminants (*p*-value < 0.001). This result reflects that these species are not equally exposed to the bacterium, as previously suggested [35,39,40], and may denote independent epidemiological cycles of *C. burnetii* in the domestic and wild ruminants in the study area. Regarding the small ruminants, the exposure risk to *C. burnetii* was not homogeneous, being 3.1 times higher in goats than in sheep. Similar findings were found in the Canary Islands [33], an insular region with a high incidence of Q fever in humans in Spain, thus highlighting the key role that goats play in the epidemiology of *C. burnetii* in this country. In this respect, an epidemic outbreak of Q fever in humans has recently been reported in northern Spain, with goats being identified as the most likely source of infection [58]. Likewise, the most significant Q fever outbreak reported to date in Europe was epidemiologically linked with this small ruminant species, resulting in an estimated 40,000 infected people and over 4000 reported cases during four successive years in The Netherlands [59,60].

Small ruminants from farms reporting reproductive disorders in primiparous females showed a significantly higher exposure to *C. burnetii*. Multiparous animals can acquire certain protections against Q fever through previous contact with the bacterium [61]. Our results underline the need to prevent young females from coming into contact with *C. burnetii* before parturition. Thus, segregating small ruminant nulliparous females from older ones at the time of their first calving could be an additional strategy to reduce new infections [62]. This approach should be combined with other measures, such as vaccination, which has been shown to effectively reduce *C. burnetii* shedding in infected herds [18].

At least one seropositive animal was detected in 96.2% of the small ruminant flocks sampled, which indicates a wide distribution of *C. burnetii* in these farms in southern Spain. However, the spatial distribution of seropositive individuals was not homogeneous among the provinces (Table S1). The geographical differences could be related to climatic factors, the presence of neighboring small ruminant farms, the animal census in farms, or the presence and density of ticks, among others [63,64]. Thus, previous studies have indicated that the lowest mean annual rainfall was associated with exposure to *C. burnetii* due to the greater likelihood of environmental dust production from dry soils [65,66]. In this sense, the highest seroprevalence being found in Almeria (72.2%) (eastern Andalusia), the area with the lowest mean annual rainfall, and the lowest seroprevalence being observed in Cádiz (21.7%) (western Andalusia), the area with the highest annual rainfall in the study area [67], support this hypothesis (Table S1).

## 5. Conclusions

Our results indicate the widespread circulation of *C. burnetii* in small domestic ruminant farms in southern Spain, which could be of animal and public health concern. These species, especially goats, may act as important reservoirs of *C. burnetii* in the study area. Contrary, the low seroprevalence found in the wild ruminant species denotes a limited risk of *C. burnetii* exposure in their populations and suggests independent epidemiological cycles between domestic and wild ruminants. Implementing integrated surveillance programs and risk-based control strategies in target species could reduce the risk of the transmission of *C. burnetii* to other sympatric species, including humans. Further studies in different epidemiological scenarios outside the study area are necessary to confirm that our results are generalizable within the context of the Iberian Peninsula.

## Figures and Tables

**Table 1:** Generalized estimating equations analysis of risk factors associated with exposure to *Coxiella burnetii* in small ruminants in southern Spain.

Variable	Categories	*p*-Value	OR (95% CI)
Species	Goat Sheep	0.028 ^a^	3.1 (1.1–8.5) ^a^
Reproductive disorders in primiparous females	Yes No	0.001 ^a^	4.3 (1.8–10.5) ^a^

^a^ Reference category.

## Data Availability

The data included in this study are available on request from the corresponding author.

## Supplementary Materials

The following supporting information can be downloaded at https://www.mdpi.com/article/10.3390/ani14213072/s1, Table S1: Distribution of explanatory variables associated with *Coxiella burnetii* seropositivity in small ruminants in southern Spain; Table S2: Distribution of the seroprevalence against *Coxiella burnetii* in wild ruminants in southern Spain and results of the bivariate analysis. Supplementary information: Epidemiological questionnaire.
