# Supplementary material for: Seroepidemiology of Coxiella burnetii in Domestic and Wild Ruminant Species in Southern Spain
Source: Animals (Basel). 2024 Oct 24;14(21):3072. doi: 10.3390/ani14213072 (PMC11545245; doi:10.3390/ani14213072)
Supplement: Supplementary file 1 [file animals-14-03072-s001.zip › animals-3246180-supplementary.pdf]

Table S1. Distribution of explanatory variables associated with *Coxiella burnetii* seropositivity in small ruminants in southern Spain.

Variable	Categories	No. Positives/ Overall*	Seroprevalence (%)	*p*-value
Species	Sheep<br>Goat	156/390<br>227/390	40.0<br>58.2	< 0.001
Sex	Female<br>Male	350/697<br>3/8	50.2<br>37.5	0.361
Breed	Purebred<br>Crossbred	123/275<br>258/496	44.7<br>52.0	0.031
Province	Almeria<br>Cadiz<br>Cordoba<br>Granada<br>Huelva<br>Jaen<br>Malaga<br>Seville	65/90<br>13/60<br>70/135<br>67/120<br>27/75<br>19/60<br>75/120<br>47/120	72.2<br>21.7<br>51.9<br>55.8<br>36.0<br>31.7<br>62.5<br>39.2	< 0.001
Production system	Meat<br>Milk<br>Mixed	134/315<br>69/120<br>154/270	42.5<br>57.5<br>57.0	< 0.001
Management system	Extensive<br>Intensive<br>Semiextensive	95/270<br>51/105<br>228/390	35.2<br>48.6<br>58.5	< 0.001
Natural mating	No<br>Yes	8/15<br>350/690	53.3<br>50.7	0.525
Census of small ruminants per farm	≤238<br>239-550<br>≥ 551	100/270<br>151/255<br>132/255	37.0<br>59.2<br>51.8	< 0.001
Presence of dogs	No<br>Yes	0/15<br>383/765	0.0<br>50.1	< 0.001
Presence of cats	No<br>Yes	114/255<br>269/525	44.7<br>51.2	0.051
Presence of cattle	No<br>Yes	322/630<br>48/120	51.1<br>40.0	0.016
Presence of pigs	No<br>Yes	351/660<br>32/105	53.2<br>30.5	< 0.001
Presence of equines	No<br>Yes	238/510<br>133/255	46.7<br>52.2	0.088
Presence of poultry	No<br>Yes	208/435<br>175/345	47.8<br>50.7	0.231
Presence of wild boar	No<br>Yes	224/405<br>159/375	55.3<br>42.4	< 0.001
Presence of red deer	No<br>Yes	304/555<br>79/225	54.8<br>35.1	< 0.001
Presence of fallow deer	No<br>Yes	379/765<br>4/15	49.5<br>26.7	0.066

Presence of mouflon	No<br>Yes	352/735<br>31/45	47.9<br>68.9	0.005
Presence of badgers	No<br>Yes	305/570<br>78/210	53.5<br>37.1	< 0.001
Distance to nearest farm (m)	0<br>1-1,000<br>≥ 1,000	104/240<br>170/285<br>55/165	43.3<br>59.6<br>33.3	< 0.001
Cattle in neighboring farms	No<br>Yes	242/480<br>93/240	50.4<br>38.8	0.002
Sheep in neighboring farms	No<br>Yes	160/285<br>175/435	56.1<br>40.2	< 0.001
Goats in neighboring farms	No<br>Yes	151/345<br>184/375	43.8<br>49.1	0.089
Disinfection baths (entry)	No<br>Yes	279/480<br>29/120	58.1<br>24.2	< 0.001
Perimeter livestock fencing	No<br>Yes	98/150<br>230/480	65.3<br>47.9	< 0.001
Dung removal system	No<br>Yes	10/30<br>298/555	33.3<br>53.7	0.023
Use of manure as fertilizer	No<br>Yes	45/120<br>276/495	37.5<br>55.8	< 0.001
Presence of sanitary lazaretto	No<br>Yes	21/60<br>296/555	35.0<br>53.3	0.005
Quarantine of replacement animals	No<br>Yes	221/435<br>87/165	50.8<br>52.7	0.371
Carcass container	No<br>Yes	199/435<br>120/165	45.7<br>72.7	< 0.001
Carcass removal system	No<br>Yes	38/75<br>290/555	50.7<br>52.3	0.446
Parking outside the farm	No<br>Yes	144/300<br>177/315	48.0<br>56.2	0.025
Carcass vehicle is allowed to enter the farm perimeter	No<br>Yes	222/420<br>97/180	52.9<br>53.9	0.444
Indirect contact with other farms (machinery)	No<br>Yes	292/555<br>29/60	52.6<br>48.3	0.310
Indirect contact with other farms (staff)	No<br>Yes	159/330<br>224/450	48.2<br>49.8	0.356
Indirect contact with other farms (pastures)	No<br>Yes	311/630<br>72/150	49.4<br>48.0	0.417
Indirect contact with other farms (troughs)	No<br>Yes	336/690<br>47/90	48.7<br>52.2	0.302
Presence of rodents	No<br>Yes	102/210<br>202/375	48.6<br>53.9	0.126

Rodent control	No<br>Yes	91/210<br>206/360	43.3<br>57.2	0.001
Cleaning protocol	No<br>Yes	64/135<br>234/450	47.4<br>52.0	0.201
Disinfection protocol	No<br>Yes	67/150<br>231/435	44.7<br>53.1	0.046
Reproductive disorders in adults	No<br>Yes	109/225<br>141/210	48.4<br>67.1	< 0.001
Reproductive disorders in primiparous females	No<br>Yes	134/285<br>103/120	47.0<br>85.8	< 0.001
Vaccination against enterotoxemia	No<br>Yes	106/225<br>277/555	47.1<br>49.9	0.265
Vaccination against pasteurellosis	No<br>Yes	322/660<br>61/120	48.8<br>50.8	0.377
Vaccination against contagious agalactia	No<br>Yes	259/465<br>124/315	55.7<br>39.4	< 0.001

* Missing values omitted.

Table S2. Distribution of the seroprevalence against *Coxiella burnetii* in wild ruminants in southern Spain and results of the bivariate analysis.

Variable	Categories	No. Positives/ Overall*	Seroprevalence (%)	*p-value*
Species	Mouflon<br>Red deer<br>Iberian ibex	2/110<br>6/390<br>1/105	1.8<br>1.5<br>1.0	0.863
Age	Juvenil<br>Subadult<br>Adult	0/139<br>3/174<br>6/290	0.0<br>1.7<br>2.1	0.244
Sex	Female<br>Male	5/255<br>4/349	2.0<br>1.1	0.313
Hunting season	2015-2016<br>2016-2017<br>2017-2018<br>2018-2019<br>2019-2020<br>2020-2021<br>2021-2022<br>2022-2023	1/92<br>0/99<br>6/129<br>0/103<br>1/98<br>1/42<br>0/23<br>0/19	1.1<br>0.0<br>4.7<br>0.0<br>1.0<br>2.4<br>0.0<br>0.0	0.073
Province	Cordoba<br>Jaen<br>Seville	1/238<br>8/265<br>0/102	0.4<br>3.0<br>0.0	0.022

*Missing values omitted.

Supplementary information: Epidemiological questionnaire

A. General data

Date: __________

A.1. Name: ______________________________

A.2. E-mail: ____________________ **A.3. Phone: ______________________**

B. General and production farm data:

B.1 Herd ID: _________________________________

B.2 Location (coordinates): X__________________Y______________________

B.3 Municipality: __________________ **Province: ______________________**

B.4 Production system: Meat □ Milk □ Mixed □

B.5 Management system: Extensive □ Intensive □ Semi-extensive □

B.6 Natural mating: Yes □ No □

B.7 Artificial insemination: Yes □ No □

B.8 Type of replacement: External □ Internal □

B.9 Census of small domestic ruminants:

	Sheep	Goat
Male		
Female		
TOTAL		

B.11 Presence of dogs: Yes □ No □
(Number):
B.12 Presence of cats: Yes □ No □
(Number):

B.13 Presence of other domestic or wild species:

Pig	Cattle	Equines	Poultry	Wild boar	Red deer	Fallow deer	Mouflon	Badger	Others

B.14 Distance to nearest ruminant farm: _________ meters.

C. Biosecurity and healthy parameters:

C.1 Disinfection baths (entry) available: Yes □ No □

C.2 Perimeter livestock fence around the farm: Yes □ No □

C.3 Dung removal system: Yes □ No □

C.4 Use of manure as fertilizer: Yes □ No □

C.5 Presence of sanitary lazaretto: Yes □ No □

C.6 Quarantine of replacement animals: Yes □ No □

C.7 Proper disposal of dead animals: Yes □ No □

C.8 Carcass removal system: Yes □ No □

C.9 Parking outside the farm: Yes □ No □

C.10 Livestock transport vehicles enter the farm perimeter: Yes □ No □

C.11 Carcass vehicle is allowed to enter the farm perimeter: Yes □ No □

C.12 Presence of other domestic ruminant species in neighboring farms: Yes □ No □

(Species):

C.13 Possibility of indirect contact with other farms:
Machinery □ Staff □ Pastures □ Troughs □ Other: ______

C.14 Presence of rodents in the farms: Yes □ No □

C.15 Biosecurity measures:

Protocols	Yes	No
Cleaning		
Disinfection		
Farm use of insecticide		
Control program for rodents		

C.16 Vaccination protocol

Vaccination	Yes	No
Pasteurellosis		
Enzootic abortion		
Contagious agalactia		
Q fever		

Other vaccines: _______________

SAMPLING

	Animal ID	Laboratory ID	Age	Gender	Breed
1					
2					
3					
4					
5					
6					
7					
8					
9					
10					
11					
12					
13					
14					
15					
16					
17					
18					
19					
20					
21					
22					
23					
24					
25					
26					
27					
28					
29					
30					
